# Nutritional Components and Digestibility Profiles of Some Potential Plant-Based Protein Sources

**DOI:** 10.3390/foods14101769

**Published:** 2025-05-16

**Authors:** Paul Ndubuisi Anyiam, Suphat Phongthai, Samart Sai-Ut, Passakorn Kingwascharapong, Young Hoon Jung, Wanli Zhang, Saroat Rawdkuen

**Affiliations:** 1 Innovative Food Science and Technology Program, School of Agro-Industry, Mae Fah Luang University, Chiang Rai 57100, Thailand; 6671401002@lamduan.mfu.ac.th; 2 Department of Biochemistry, College of Natural Science, Michael Okpara University of Agriculture, Umudike, Umuahia P.M.B 7262, Abia State, Nigeria; 3 Division of Food Science and Technology, Faculty of Agro-Industry, Chiang Mai University, Chiang Mai 50100, Thailand; su.phongthai@gmail.com; 4 Faculty of Science, Burapha University, Chonburi 20131, Thailand; samarts@go.buu.ac.th; 5 Department of Fishery Products, Faculty of Fisheries, Kasetsart University, Bangkok 10900, Thailand; passakorn.ki@ku.th; 6 School of Food Science and Biotechnology, Kyungpook National University, Daegu 41566, Republic of Korea; younghoonjung@knu.ac.kr; 7 College of Food Science and Technology, Hainan University, Haikou 570228, China; zwl@hainanu.edu.cn; 8 Unit of Innovative Food Packaging and Biomaterials, School of Agro-Industry, Mae Fah Luang University, Chiang Rai 57100, Thailand

**Keywords:** amino acid, biological qualities, compositions, digestibility, plant-based protein

## Abstract

Background: The dominance of soybeans as the primary plant protein source has hindered the exploration of potential sources, limiting dietary diversity and innovation. Objective/Methods: This study evaluated six plant protein sources—mung bean (MB), bambara bean (BN), jack bean (JB), sesame seed (SS), moringa seed (MS), and rice bran (RB)—compared to soybean (SB) for their chemical composition and biological qualities using standard methods. Results: Protein composition (14.98–30.29 g/100 g), fiber (2.90–8.18 g/100 g), and fat (5.19–33.30 g/100 g) varied across plants. Bulk density (0.49–0.74 g/mL), swelling capacity (0.25–0.55%), and yellowness (13.07–38.76) were comparable to SB. Electropherograms showed major protein bands at 20, 48, 75, and 100 kDa across plant proteins under non-reducing conditions. Phytate levels were highest in RB, while MS showed lower tannic acid composition (6.64 mg/100 g) compared to SB. Protein solubility (24.64–45.65%) increased with pH, while in vitro protein digestibility (74.86–87.64%) varied and was slightly below SB (91.07%); however, a similar pattern of protein digestion was observed under no reducing condition. MS and BN contained 31.17% and 42.47% of total essential amino acids with PDCAAS values of 41.42% and 58.46%, respectively. Conclusions: Overall, MS and BN exhibited superior potential as sustainable protein sources, showing properties comparable to soybean.

## 1. Introduction

Beyond meeting basic caloric needs, protein remains the second-most vital nutrient for human growth and development [1]. Protein from animal sources is considered complete due to its high quality, superior functionality, and digestibility [2]. However, animal protein sources often raise concerns regarding environmental sustainability, resource consumption, ethical challenges, and health implications [2,3]. In contrast, plant-based proteins offer a sustainable solution and have gained recognition due to their lower environmental footprint, affordability, widespread availability across the globe, and potential health benefits [4]. For example, plant-based proteins are closely associated with lowering low-density lipoprotein cholesterol, type 2 diabetes, cardiovascular conditions, and oxidative stress [5]. However, their protein quality is generally lower than that of animal proteins because they contain anti-nutritional factors that reduce the bioavailability of nutrients. They also have a lower amount of one or more essential amino acids relative to human requirements, which may limit their use in the food industry. Addressing these limitations requires advancements in plant protein ingredient development to enhance the production of plant-based foods.

Plant proteins are emerging as key ingredients for developing innovative products and strengthening food security. As a result, the global market for plant-based food protein is rapidly growing [6]. For instance, the global plant-based meat production is expected to reach around 25 million metric tons by 2030 [7], driving an increased demand for plant protein production. This surge in demand is driven by growing consumer awareness of their health benefits, along with concerns about the environmental and ethical impacts of animal proteins [2]. Soybeans (*Glycine max*) remain the primary commercial source of plant proteins for plant-based food processing due to their high protein compositions (28–52%) and versatility in food applications [7,8]. Soybean seeds provide all eight essential amino acids, and their oil consists of more than 80% unsaturated fatty acids [7,9]. However, dependence on soybeans presents risks, as they are sensitive to climate change, with rising temperatures and unpredictable weather patterns affecting their yield in specific regions [10,11]. Additionally, soybean, a widely commercialized crop often cultivated in monoculture, is known to cause allergic reactions in some consumers [7]. These limitations highlight the necessity for exploring a wider array of drought-tolerant alternative plant protein sources that have similar or better biological qualities as a soybean substitute in food processing.

Mung bean (*Vigna radiata*) is primarily grown in Asia for its short growth cycle (70–90 days), high average yield (400 kg/ha), drought tolerance, and nitrogen-fixing ability [3]. It is well-regarded for its protein-rich (18–33%) and essential amino acid compositions [7,9]. Studies have also shown that mung beans are a valuable source of essential minerals like calcium, potassium, and iron, as well as vitamins such as A, E, and C [9]. In many sub-Saharan African countries, mung bean is primarily utilized for its starch and as animal feed, with limited focus on developing its protein potential for food applications. Dimopoulou et al. [9] suggested that increased consumption of mung beans could help in preventing and managing metabolic disorders, including diabetes, obesity, and cardiovascular conditions. Bambara beans (*Voadzeia subterranea*) yield 18–28% protein with a balanced amino acid profile and thrive well in drought-prone and poor soils [12]. Bambara beans vary in color, depending on the soil and growing location [13]. They are used in traditional African dishes, including soups and porridges, snacks with palm kernel, and fried bean balls (*akara*). Despite its benefits, value-added products of bambara bean remain limited in commercial markets. Jack bean (*Canavalia ensiformis*) is a hardy legume cultivated in Asia, Africa, and the Americas [14]. It enhances soil fertility and withstands harsh climates, offering 20–39% protein and low fat [14,15]. Jack beans are traditionally used as animal feed, though in some areas they are eaten after thorough processing to reduce anti-nutrients. Sesame (*Sesamum indicum* L.) seeds contain 20–34% protein and valuable amino acids [16]. Sesame seeds are primarily used for oil production due to their high oil content. The oil extraction process yields a protein-rich meal, which is typically used as animal feed or discarded. Although the demand for sesame oil is increasing, sesame meal has not been fully utilized. *Moringa oleifera* (family: *Moringaceae*) is a fast-growing, edible tropical plant cultivated across Africa, Asia, and the Americas. Traditionally, *Moringa oleifera* (particularly its leaves) has been consumed as a vegetable and used in folk medicine to treat various ailments in many African and Asian communities. Its seeds are rich in essential amino acids, providing 28–45% protein with significant health benefits [17,18]. Rice bran (*Oryza sativa*), which is derived from the outer layer of rice during the milling process, provides 10–16% protein with numerous health benefits [5,19]. Rice bran has been traditionally used as not only livestock feed but also in some cultures for oil extraction due to its high oil content. While integrating these plant proteins into food systems could bolster global protein supplies and encourage plant-based food production, their commercial availability remains limited in the global market. Highlighting their properties and protein quality could enhance their use in food processing.

Biological qualities of food protein evaluation typically involve assessing essential amino acid supply and digestibility through animal studies. Recent studies have employed several methods to evaluate the protein quality of plant-based foods [20,21]. Among these methods, the digestible indispensable amino acid score (DIAAS) and the protein digestibility-corrected amino acid score (PDCAAS) are valuable methods for assessing how well proteins supply essential amino acids to meet human nutritional needs [22]. The PDCAAS is the most commonly used method due to its cost-effectiveness, efficiency, and direct association with human protein requirements. However, FAO, in its last experts’ reports, proposes DIAAS as a more precise method. Authors proposed that using the in vitro PDCAAS method for assaying protein quality is a good alternative that does not depend on animal experimentation (i.e., rats or pigs) [20]. Despite their nutritional benefits, the use of these potential plant protein sources in food production remains low, with only a limited number of value-added products currently available in the commercial market. This may be due to a lack of knowledge about their composition, characteristics, and potential applications. The properties and quality of plant-based protein sources can differ greatly depending on the geographical region where they are grown and farming practices. Given the diverse varieties of plant protein sources across various regions, studying their biological properties is essential to expanding their potential utilization. Therefore, this study was aimed to comparatively analyze the chemical composition, biological qualities, and protein digestibility pattern of six alternative plant protein sources grown in Thailand. By offering valuable insights into their potential as plant-based protein alternatives, this research contributes to the development of sustainable protein options. The findings could facilitate the incorporation of these plant sources into food products and serve as a reference for future studies.

## 2. Materials and Methods

### 2.1. Plant Materials and Chemicals

**Figure 1:**
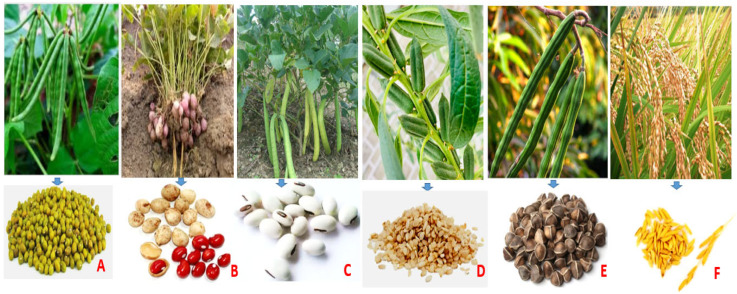
Plant protein sources—trees and seeds: (**A**) green mung bean, (**B**) red bambara bean, (**C**) creamy-white jack bean, (**D**) white-creamy sesame seed, (**E**) moringa seed, and (**F**) rice bran.

Approximately 5 kg each of mature, fresh samples of mung bean, bambara bean, jack bean, sesame seed, moringa seed, and rice bran were sourced from a local market in Mueang district, Chiang Rai Province, Thailand. The plant material varieties used in this study, as shown in Figure 1, were selected based on their useful agronomic traits and availability within the study location. Soybeans, used as a reference, were purchased from a supermarket in the same area. The equipment and chemicals used in this study, including methyl red, Tris(hydroxymethyl) aminomethane (THAM), sodium dodecyl sulfate, glacial acetic acid, Coomassie blue R-250, methanol, and others, were supplied by RCI LabScan Ltd., Bangkok, Thailand. Additional reagents such as pancreatin (EC Number: 232-468-9), pepsin (EC Number: 3.4.23.1), and bovine serum albumin (BSA) were sourced from Sigma-Aldrich Chemical Co., St. Louis, MO, USA. The pre-stained standard protein marker was provided by Bio-Helix Co. Ltd., Taipei City, Taiwan. All chemicals and reagents used were of analytical grade.

### 2.2. Sample Processing

All plant samples were removed from their pods (except sesame and rice bran, which have no pod) and washed with tap water to remove dirt. The samples were visually screened, and defective and immature seeds and beans were discarded. They were then manually dehulled to separate the coats and dried in an oven (Memmert-A55/33, Schwabach, Germany) at 45 °C for 36 h, ground into powder with an electric blender (RT-04A, Rong Tsong Tech. Co., Taichung, Taiwan), and passed through a 2 mm mesh for fine consistency. The resulting powder was labeled and kept in airtight containers at 4 °C for later analysis. These processing conditions were chosen to minimize heat-induced protein denaturation and preserve the biological properties of the plant proteins, making the resulting powder suitable for further analysis.

### 2.3. Chemical Composition, Anti-Nutritional Compound, and Proximate Analysis

#### 2.3.1. Proximate Analysis

The proximate analysis on each of the processed plant materials was conducted in triplicate following the method described in AOAC [23] methods. The dry ashing method (AOAC-920.15) at 550 °C was used for the determination of ash, while the oven-dry method (AOAC-950.46) was adopted for moisture determination. The Kjeldahl method (AOAC-954.01) was used for protein composition evaluation using a nitrogen-to-protein conversion factor of 6.25. Fat content was measured using the Soxhlet method (AOAC-960.39) with petroleum ether, while crude fiber was evaluated by digestion method (AOAC-991.43) using hot sulfuric acid and hot sodium hydroxide. Carbohydrate composition was calculated by determining the percentage difference from the other components.

#### 2.3.2. Determination of Tannin Content

The tannin composition in each test plant material and the reference sample was determined according to the method described by Makkar et al. [24] using Folin–Denis reagent. This method involves the preparation of a standard curve of pure tannic acid. The sample (1 g) was extracted with 40 mL of 10% methanol and then filtered, and the volume was adjusted to up to 50 mL. Aliquots (1 mL) of the extract were placed in a volumetric flask, 10 mL of 35% sodium carbonate reagent was added, followed by adjustment of the volume to 100 mL. The absorbance was measured at 760 nm, and the tannin content in each sample was determined in triplicate assay.

#### 2.3.3. Determination of Phytic Acid Content

Phytic acid composition in the plant samples was determined using the method of Reddy et al. [25]. A 2 g sample was placed into a beaker and soaked with 100 mL of 2% HCl for 5 h, then filtered through Whatman No. 1 filter paper. Next, 25 mL of the filtrate was transferred to a conical flask, and 5 mL of 0.3% potassium thiocyanate solution was added. The mixture was titrated with a standard 1.05% w/v FeCl_3_ solution. The endpoint was reached when a persistent brownish-yellow color appeared for 5 min. The molar ratio of Fe to phytate is 1:1, and the amount of phytate was calculated using Equation (1).

(1) $$\mathrm{Concentration}\;\mathrm{of}\;\mathrm{phytate}\;(\mathrm{mg}/g)\;=\;\frac{Titre\;value\times 0.064}{100\times sample\;weight(g)}$$

#### 2.3.4. Determination of Oxalic Acid Content

The oxalic acid content in each plant sample was determined following the method outlined by Anyiam et al. [26]. A 1.0 g sample was added to a 100 mL conical flask containing 75 mL of 3.0 M HCl. The mixture was stirred periodically with a magnetic stirrer for one hour before being filtered through Whatman No. 1 filter paper. A 25 mL aliquot of the filtrate was then titrated with a hot (90 °C) 0.1 N KMnO_4_ solution until a faint pink color appeared and remained for at least 30 s. The oxalic acid concentration was calculated using Equation (2).

1 mL of 0.1 KMnO_4_ = 0.006303 g of Oxalate (2)

### 2.4. Physicochemical Properties Determination

#### 2.4.1. Color Attribute Determination

The color attributes of the processed samples were determined at three randomly chosen spots following the method reported by Ramatsetse et al. [27] using a Hunter-Lab instrument (Cox-2339, Reston, VA, USA). The chromameter works by determining the color based on the blue, red, and green color components of the light absorbed by the sample. The instrument was first calibrated using black and white tiles to measure color parameters, including L* (lightness), a* (redness), and b* (yellowness). The values obtained were used to calculate the whiteness (WI), yellowness (YI), and change in color (Δ*E*) of each sample compared with soybean using Equations (3), (4), and (5), respectively, as follows:

(3) $$\mathrm{Whiteness}\;\mathrm{index}\;=\;100-\sqrt{(100-{L)}^{2}+{\left(a\right)}^{2}+{\left(b\right)}^{2}}$$

(4) $$\mathrm{Yellowish}\;\mathrm{index}\;=\;\frac{142.86b}{L}$$

(5) $$\Delta E=\sqrt{(\Delta {L)}^{2}+(\Delta {a)}^{2}+(\Delta {b)}^{2}}$$

#### 2.4.2. Bulk Density Determination

Bulk density of processed test and reference samples was determined by following the method described in Ramatsetse et al. [27]. A 20 g sample was placed into a pre-weighed 100 mL graduated cylinder and compacted by gently tapping the cylinder on a benchtop ten times from a height of 5–8 cm. This process was repeated until no further change in the sample’s volume (or height) was observed. The final volume of the test sample was measured, and the weight of the sample per unit volume was calculated in triplicate. The bulk density was determined using Equation (6), and the result was expressed in g/mL:

(6) $$\mathrm{Bulk}\;\mathrm{density}\;(g/\mathrm{mL})\;=\;\frac{Weight\;of\;sample\;(g)}{Volume\;of\;sample\;(mL)}$$

#### 2.4.3. Water Activity Determination

The water activity (Aw) measurement of each sample was determined by using the water activity meter (2108018, Novasina, AG, Lachen, Switzerland). The measurement cell was calibrated, and each test sample was placed individually into the sample containers and positioned inside the analyzer. The protective filter of the cell was then closed. The water activity reading was monitored until stable, and the values were recorded. The test was performed in triplicate for each sample to ensure accuracy.

#### 2.4.4. Swelling Capacity Determination

Swelling capacity (SC) of each sample was performed in triplicate determinations using the procedure explained by Ramatsetse et al. [27]. A 10 g sample of each dried powder was placed into a 300 mL measuring cylinder, and the initial volume was recorded. The powder was then added to approximately 150 mL of distilled water and allowed to sit for 4 h. After swelling, the final volume was measured, and the swelling capacity was calculated using Equation (7).

(7) $$\mathrm{Swelling}\;\mathrm{capacity}\;(\%)\;=\;\frac{Final\;volume-Initial\;volume}{initial\;volume}\times 100$$

#### 2.4.5. Protein Solubility Evaluation

The protein solubility of the samples was determined as a function of pH (2.0–10.0) following the method described by Wang et al. [28]. Sample (1 g) was dispensed in 50 mL distilled water, and the pH was adjusted between 2 and 10 with 0.1 M NaOH or HCl using a pH meter (Mettler-Toledo, LE438, Greifensee, Switzerland). The suspension was vortexed for 30 min at ambient temperature and then centrifuged at 10,000× *g* for 10 min. The supernatant was collected, and the soluble protein was determined using the biuret method at 540 nm absorbance (UV-Visible-BIC99877, Biochrom/Libra, Cambridge, UK). The experiment was performed in triplicate determinations, and the percentage of soluble protein was calculated by using Equation (8) and plotted against the corresponding pH values.

(8) $$\%\;\mathrm{Protein}\;\mathrm{solubility}\;=\;\frac{Protein\;in\;the\;supernatant}{Total\;protein\;in\;sample}\times 100$$

### 2.5. Protein Quality and Digestibility Determination

#### 2.5.1. In Vitro Gastrointestinal Digestion Assay

In vitro digestion of protein in each plant material was assessed in two phases by following the sequential digestion method outlined by Di et al. [29] and Rawdkuen et al. [30]. Protein solution (1 g/100 mL, pH 1.5) was mixed with pepsin (enzyme-to-protein ratio of 1:100 g/g) and gently stirred at 37 °C for 120 min. Sampling was performed at 0, 30, 60, and 120 min by taking 1 mL of the mixture, and pepsin activity was stopped by adding 1 mol/L NaOH. For pancreatin digestion, pancreatin (enzyme-to-protein ratio of 1:100 g/g, pH 8) was added to the neutralized pepsin-digested mixture. Sampling was performed in the same manner as for pepsin digestion and until 240 min at 37 °C. To stop pancreatin digestion, the solutions were heated in boiling water for 10 min. The mixture was centrifuged at 10,000× *g* for 10 min. Then, the protein content in the supernatant was determined by the Biuret method. SDS-PAGE was used to monitor the polypeptide hydrolysis of proteins at different time intervals (0–240 min) during pepsin and pancreatin digestion. The percentage of in vitro protein digestibility (IVPD) was calculated using Equation (9) [29].

(9) $$\mathrm{IVPD}\;(\%)\;=\;\frac{P1-P2}{P1}\times 100$$

where P1 is the initial protein content in the sample and P2 is the protein content after in vitro digestion.

#### 2.5.2. Electrophoresis

Plant sample (2 g) was mixed with 5% SDS solution (18 mL), homogenized (IKA 372000I T18, Ultra-Turrax, Wilmington, NC, USA) at 10,000 rpm for 2 min, and then heated at 85 °C for 1 h to facilitate protein extraction. The protein solution was allowed to cool to room temperature and was centrifuged (10,000× *g* for 5 min). The protein content was measured using the Biuret method. The molecular weight of protein was determined with SDS-PAGE according to the method of Laemmli [31], using 12% separating gel and 4% stacking gel. Each protein sample (4 mg/mL) was mixed with loading buffer solution at the ratio of 1:1 (*v*/*v*). For non-reducing conditions, the samples were mixed with the sample buffer (0.5 mol/L Tris–HCl, pH 6.8, 0.5 g/100 bromophenol blue, 10% glycerol, and 2% SDS). For reducing conditions, beta-mercaptoethanol was included in the sample buffer. The mixture was heated at 95 °C for 5 min, then cooled to room temperature. An aliquot (4 μL) containing 15 μg protein was loaded onto the 4–12% gradient gels and subjected to separation at 15 mA/gel in an electrophoresis buffer tank filled with running buffer using Mini Protean Tetra Cell units (Bio-Rad Laboratories, Inc., Richmond, CA, USA). The gel was stained overnight with Coomassie Brilliant Blue R-250 while gently shaking at 50 rpm. It was then de-stained with de-staining solutions I and II (methanol–acetic acid–water) until the background cleared, dried, and photographed under white light. A molecular weight protein marker (11–245 kDa) was used as a standard to estimate the proteins’ molecular weight.

#### 2.5.3. Amino Acid Analysis

The amino acid profile was determined for moringa seed and bambara bean, as they showed the best performance in both chemical composition and protein digestibility. The amino acid composition was determined at the Central Laboratory Co., Ltd., Chiang Mai, Thailand, using an in-house protocol according to the AOAC [23] method (number: 994.12). The amino acids were extracted from each sample by hydrolysis with 6 M HCl. The hydrolysates were diluted with a sodium citrate buffer, and the pH was adjusted to 2.2. Individual amino acid components were separated and identified by using ion exchange chromatography and determined by reaction with ninhydrin with photometric detection at 570 nm (440 nm for proline). The content of each amino acid was expressed in g/100 g of protein.

#### 2.5.4. Estimation of Biological Quality of Protein

The proportion of essential amino acids to total amino acids (E/T), essential amino acid index (EAAI), amino acid score (AAS), and in vitro protein digestibility-corrected amino acid score (IV-PDCAAS) were used to assess the biological quality of protein from each of the analyzed plant protein sources following the method described in Samaei et al. [32] by using the expressions in Equations (10) and (11), respectively.

(10) $$E/T\;(\%)\;=\;\frac{Sum\;of\;essential\;amino\;acids}{Total\;amount\;of\;amino\;acids}\times 100$$

(11) $$\mathrm{EAAI}\;(\%)\;=\;\sqrt[{9}]{\left\{\frac{(Lys\times Thr\times Val\times Met\times Ile\times Leu\times Phe\times His\times Trp)a}{(Lys\times Thr\times Val\times Met\times Ile\times Leu\times Phe\times His\times Trp)b}\right\}}\times 100$$

where ‘*a*’ in Equation (11) represents the content of amino acids in the test sample and ‘*b*’ is the content of the same amino acids in a reference standard protein, respectively.

The obtained amino acid profile results were compared against the FAO/WHO [22] reference protein for pre-school children to calculate the AAS and the IV-PDCAAS by using Equations (12) and (13), respectively. The amino acid score is based on the amount of the first-limiting amino acid in each of the protein materials. The protein digestibility value for each sample was converted to a decimal by dividing by 100 prior to calculation of the IV-PDCAAS, and the final result was expressed as a percentage as follows:

(12) $$\mathrm{Amino}\;\mathrm{acid}\;\mathrm{score}\;(\mathrm{AAS})\;\%\;=\;\frac{mg\;of\;limiting\;Amino\;acid\;in\;test\;sample}{mg\;of\;same\;Amino\;acid\;in\;reference\;protein}\times 100$$

(13) IV-PDCAAS (%) = In−vitro protein digestibility×Lowest amino acid score

### 2.6. Statistical Analysis

All experiments were conducted in triplicate to ensure the precision and accuracy of results. Data were presented as mean ± standard deviation. Collected data were subjected to statistical analysis of variance (ANOVA) with the statistical package for the social sciences (SPSS Inc., version 22.0, Chicago, IL, USA). The least significant difference (LSD) post hoc test was used to compare means, and significance was accepted at *p* < 0.05.

## 3. Results

### 3.1. Proximate Composition and Anti-Nutritional Factors of Plant Protein Sources

The proximate analysis (Table 1) and anti-nutritional properties (Table 2) of the six plant protein varieties in this study revealed that their major components were protein, carbohydrates, and fat, while the minor components included ash, crude fiber, and moisture. All plant materials, including soybeans, had moisture content below 10%. Among the six varieties analyzed, moringa seed had the highest protein content, which was statistically comparable (*p* > 0.05) to that of soybean. In contrast, rice bran and jack bean had the lowest protein composition, both below 20%, while mung bean and sesame seeds exhibited similar protein levels. Regarding fat content, moringa seeds recorded the highest value, followed by sesame seeds. The fat content in moringa seed and sesame seed was 69.5% and 17.36% higher (*p* < 0.05), respectively, in respect to soybean. Conversely, bambara and mung beans had the lowest fat content. For fiber composition, rice bran contained significantly more fiber than soybean (*p* < 0.05), whereas the other plant sources (bambara bean, moringa seed, and mung bean) showed similar (*p* > 0.05) fiber values to soybean, except for sesame seeds and jack bean, which had significantly lower fiber content than soybean. In terms of ash content, all plant materials exhibited comparable values to soybean, except for rice bran, which had a significantly higher ash composition (*p* < 0.05). Carbohydrate analysis revealed that all plant materials had significantly (*p* < 0.05) higher carbohydrate content than soybean, with the exception of moringa seed, which recorded a significantly lower carbohydrate content when compared with soybean. Jack bean had the highest carbohydrate composition among the plant protein sources evaluated.

In terms of phytate composition (Table 2), rice bran had the highest value, significantly higher than soybean, whereas the lowest value was seen in sesame seed. The highest oxalate content was recorded in jack bean, much higher than soybean, while mung bean, bambara bean, and moringa seed had comparable values of oxalate. Bambara bean had a higher tannic acid composition than soybean (*p* < 0.05), followed by mung bean, while other plant materials recorded lower tannic acid compositions than soybean.

### 3.2. Physicochemical Properties Determination

#### 3.2.1. Bulk Density, Swelling Capacity, and Water Activity

Bulk density (BD), swelling capacity (SC), and water activity (Aw) of the plant samples are shown in Table 2. Mung bean, sesame seed, and jack bean exhibited similar BD (*p* > 0.05) with soybean, while rice bran, bambara bean, and moringa seed recorded lower BD. All test plant proteins recorded lower SC (*p* < 0.05) compared with soybean. The Aw values for all the test plant protein materials ranged from 0.38 to 0.60. Moringa seed exhibited the highest Aw value (*p* < 0.05), while rice bran had the lowest Aw value. Among all the plant protein sources assessed, the Aw values were significantly different from soybean (*p* < 0.05), except for jack bean, which showed a statistically similar Aw value (*p* > 0.05) compared with soybean.

#### 3.2.2. Color Attributes

The color attributes of the plant materials are shown in Table 3: From the result obtained, all the color attributes (L*, a*, and b*) of the plant materials were significantly different (*p* < 0.05) when compared with soybean except for sesame, which recorded a similar b*-value (yellow) with soybean (*p* > 0.05). The highest L* value (lightness) was shown in soybean and was significantly different from all test plant materials (*p* < 0.05). Rice bran and sesame seeds recorded the lowest lightness but had higher yellowness than soybean, while other plant materials recorded lower yellowness compared with soybean. Among all plant samples, only mung bean had a green tone (negative a* value). The whiteness (WI), indicating overall brightness, was highest in jack bean and lowest in rice bran. When compared with soybean, all the plant materials evaluated had higher brightness (*p* < 0.05) than soybean, except for rice bran and sesame seeds, which recorded lower values. The change in color (ΔE) compared to soybean (as reference) showed significant variations (*p* < 0.05) across the plant materials. Rice bran exhibited the highest ΔE, followed by moringa seed, while bambara bean and jack bean displayed the lowest ΔE values (*p* < 0.05) compared with soybean.

#### 3.2.3. Protein Solubility

**Figure 2:**
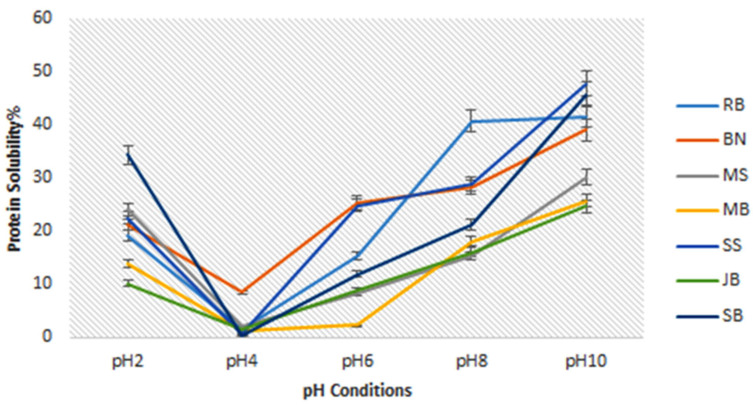
Protein solubility of plant protein sources. RB = rice bran, BN = bambara bean, MS = moringa seed, MB = mung bean, SS = sesame, JB = jack bean, SB = soybean.

The protein solubility profiles of the six plant-based protein sources at various pH values (2–10) are presented in Figure 2. The results showed that all plant proteins had similar protein solubility patterns to soybean and increased as a function of pH from pH 2–10. The minimum solubility occurred at pH 4, and the highest solubility of the protein occurred at pH 10 across all samples, except for rice bran, where the solubility was highest at pH 8 and slightly decreased at pH 10. A similar solubility pattern was observed across all samples, showing V-shaped curves. Sesame seed and rice bran recorded almost similar percentage solubility (*p* > 0.05) (47.65% and 41.56%) with soybean (45.65%) at pH 10, while the protein of jack bean, mung bean, and moringa seed recorded significantly (*p* < 0.05) lower solubility compared with soybean.

### 3.3. Protein Quality and Digestibility Determination

#### 3.3.1. Protein Patterns of Plant Proteins

**Figure 3:**
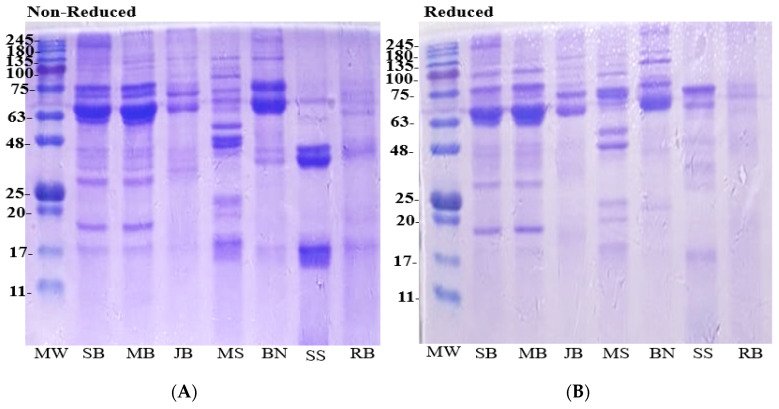
Electrophoretic patterns of plant protein sources. 12% separating gel and 4% stacking gel. 15 μg protein was loaded in each lane. MW: molecular weight. SB = soybean, MB = mung bean, JB= jack bean, MS = moringa seed, BN = bambara bean, SS = sesame, RB = rice bran. (**A**) non-reduced and (**B**) = reduced conditions.

Electrophoretic mobility of the proteins in each plant material, according to their different molecular weights, is shown in Figure 3. Under non-reduced conditions, the electropherogram revealed that the soybean has major protein bands with molecular weights ranging from approximately 20–75 kDa. A similar protein pattern was observed in mung bean (20–75 kDa), bambara bean (48–75 kDa), and moringa seed (17–100 kDa), while the major protein bands in other plant materials clearly differ from soybean. Sesame protein had lower molecular weight protein bands (17–48 kDa) under non-reducing conditions. After reduction, high-molecular-weight bands between 63 and 75 kDa appeared in sesame protein, whereas the intensity of the 17 kDa band in moringa seed and the 63–75 kDa band in bambara bean decreased and became less visible under reduced conditions.

#### 3.3.2. In Vitro Protein Digestibility Pattern

**Figure 4:**
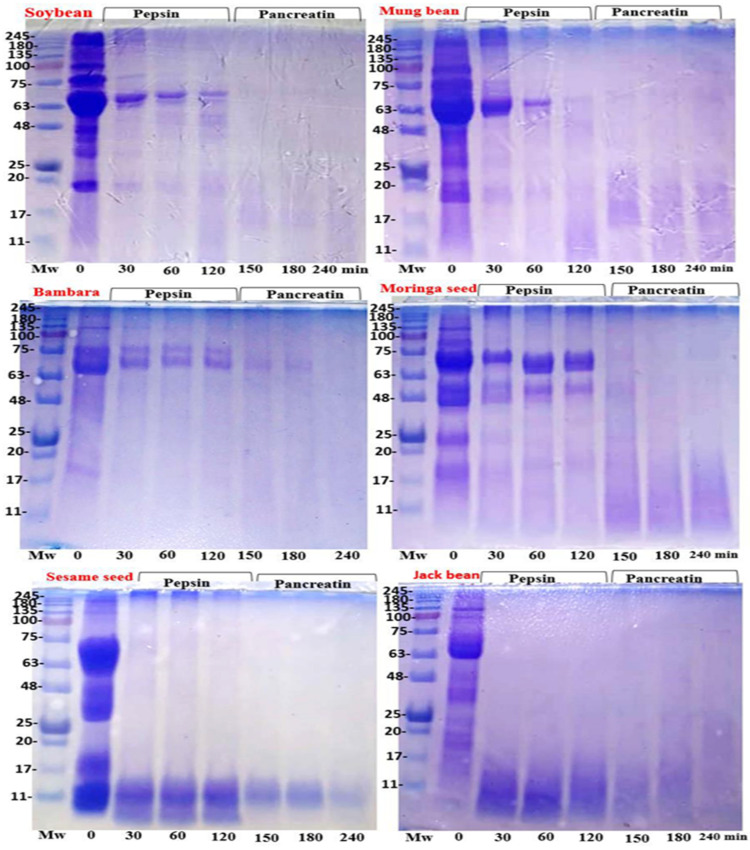
Protein digestion pattern of different plant proteins at different times. Non-reducing condition. 12% separating gel and 4% stacking gel. 15 μg of protein was loaded in each lane. MW: molecular weight. Number: time (min).

The in vitro protein digestibility (IVPD) of the plant protein sources showed that soybean exhibited the highest IVPD of 91.07 ± 2.0%. Bambara bean and mung bean showed IVPD values of 87.64 ± 3.21% and 86.46 ± 4.45%, respectively, closely comparable with soybean, while the IVPD of rice bran (84.85 ± 1.40%), moringa seed (83.19 ± 6.03%), jack bean (84.43 ± 2.96%), and sesame seed (74.86 ± 3.02%) were significantly lower (*p* < 0.05) than that of soybean. Sesame seed had the lowest in vitro digestibility in the pancreatin phase, significantly differing from soybean (*p* < 0.05). To further examine the protein digestion patterns in each plant material, SDS-PAGE was performed. Figure 4 displays the protein bands of each sample during in vitro digestion by pepsin (0–120 min) and pancreatin (120–240 min) under non-reducing conditions. Proteins with molecular weights (Mws) ranging from approximately 20–100 kDa were detected at the start of digestion (0 min) across all plant materials. Following simulated pepsin digestion for 120 min, these proteins were hydrolyzed, producing smaller fragments with reduced band intensity in all plant materials. Further digestion in simulated intestinal fluid with pancreatin (120–240 min) resulted in barely detectable protein bands in the polyacrylamide gel. Bambara bean, moringa seed, and mung bean exhibited digestion patterns similar to soybean during both the pepsin and pancreatin phases. However, sesame seed and jack bean displayed distinct digestion profiles, characterized by lower molecular weight protein bands (<17 kDa) after pepsin digestion, which almost completely disappeared during pancreatin digestion (<11 kDa) under the same conditions.

#### 3.3.3. Comparative Radar Analysis

**Figure 5:**
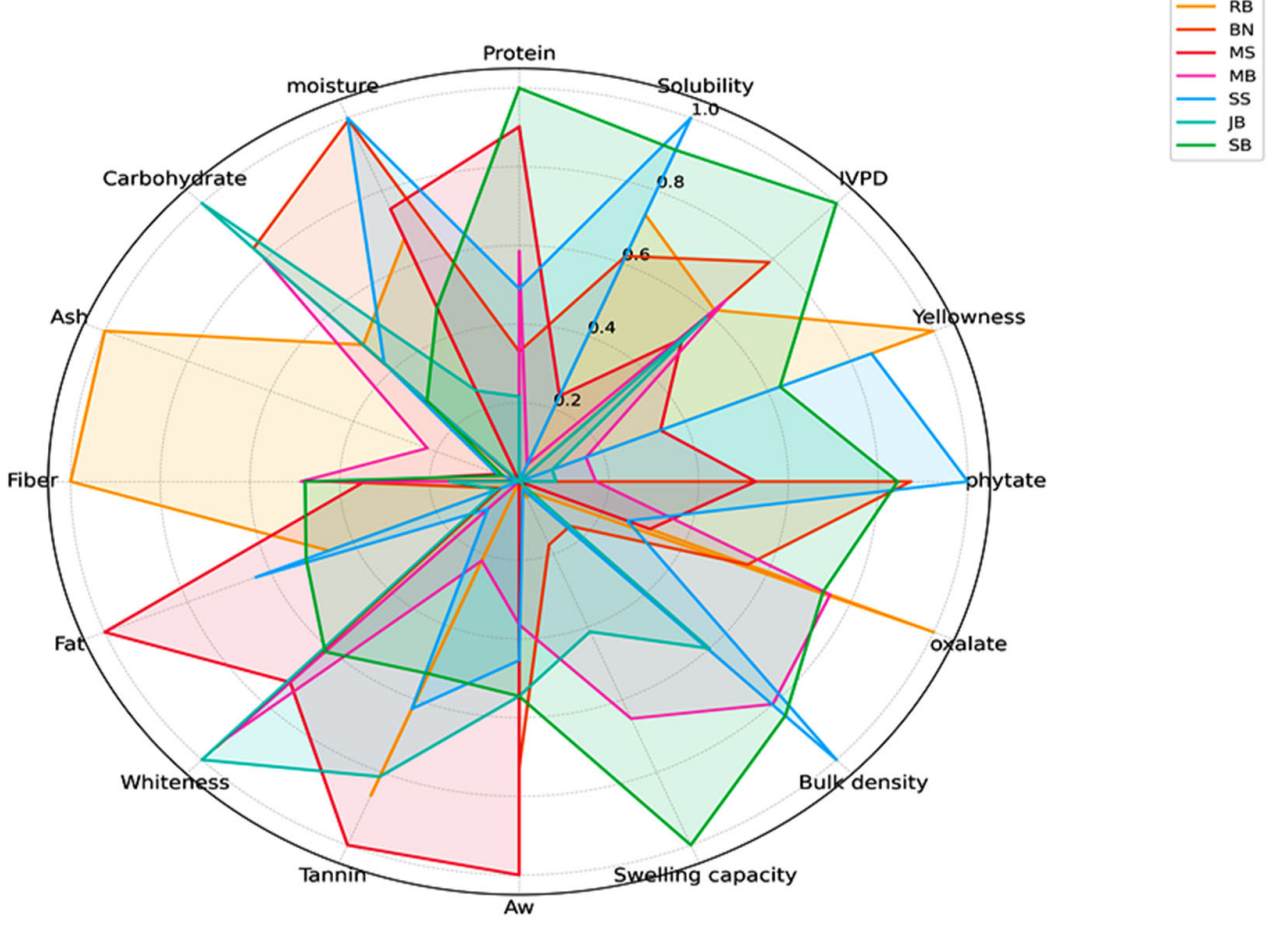
Comparative radar analysis of soybean and other plant protein sources. Note: Data on anti-nutritional factors were inverted to allow for proper interpretation.

A comparative radar plot analysis (Figure 5) was performed between all parameters obtained to show which plant protein source is closest to soybean. To enable a comparative visualization of all attributes, each attribute was normalized for proper scaling and interpretation. The selection was based on the following three primary criteria: (1) protein composition, (2) in vitro protein digestibility (IVPD), and (3) protein solubility. Other attributes, such as anti-nutritional factors and physicochemical properties, were also considered in the selection. Based on the selection criteria, moringa seed and bambara nut showed the closest similarity with soybean in terms of chemical compositions, IVPD, and solubility. Consequently, these two protein sources were selected for further evaluation of their amino acid profiles and protein quality.

#### 3.3.4. Amino Acid Composition

The amino acid composition of moringa seed and bambara bean was analyzed against the FAO/WHO reference pattern for essential amino acids in children, with results presented in Table 4. The analysis revealed that bambara bean exhibited higher total essential amino acid content and total amino acid composition compared to moringa seed. Also, the essential amino acid index of bambara bean (116%) was higher than that of moringa seed (86.26%). However, moringa seed is rich in glutamic acid, arginine, and leucine, while bambara bean predominantly contains lysine, aspartic acid, phenylalanine, and glutamic acid. Moringa seed exhibited a higher concentration of sulfur-containing amino acids (methionine and cysteine) compared to bambara bean. However, bambara bean exceeded FAO/WHO recommendations for threonine, valine, isoleucine, leucine, histidine, lysine, and tryptophan. Methionine was the first-limiting amino acid in bambara nut, with the lowest amino acid score (AAS) of 67.20%. Conversely, moringa seed met or surpassed the reference levels for threonine, valine, isoleucine, leucine, histidine, and tryptophan but had lysine as its first-limiting amino acid when compared with reference protein, with an AAS of 49.31%. However, the protein digestibility-corrected amino acid score (PDCAAS) was higher in bambara bean than for moringa seed.

## 4. Discussion

### 4.1. Proximate Compositions

Proximate composition is a key factor in assessing the nutritional quality and suitability of food materials. According to Codex Alimentarius Standards, the maximum allowable moisture content in flour is 14.5%. In all plant protein sources analyzed, including soybean, moisture levels remained below this threshold, which is a crucial factor for ensuring long-term storage stability of the plant materials. Excessive moisture content in flour can lead to increased enzyme activity, microbial growth, and the formation of lumps during food processing [15]. The variations in moisture composition across the plant protein sources, despite passing through the same processing conditions could be due to differences in their intrinsic water-binding capacity and composition of hydrophilic compounds such as polysaccharides and proteins [33]. Proteins with more hydrophilic amino acid residues can interact with water through hydrogen bonding, enhancing the ability to retain moisture. Variations in surface area, porosity, and lipid content may also have influenced the extent to which moisture is lost or retained during sample processing [34].

Proteins play crucial roles in food processing by influencing both the nutritional quality and sensory characteristics of food products. The variation in protein composition noted among the six plant protein sources compared to soybean could be related to variability in composition and mechanisms of accumulation of their storage proteins, which are governed by genetic, physiological, and environmental factors [7]. For instance, Li et al. [35] reported that the biosynthesis and accumulation of protein in soybean is tightly connected to the seed maturation and influenced by external environmental factors. Antonets et al. [36] revealed the involvement of the amyloid formation mechanism in the accumulation of storage proteins in peas, which increases with seed maturation. Plant materials like rice bran and sesame seed may prioritize oil storage mechanisms over protein synthesis, while others, like mung bean, jack bean, and bambara bean (with low fat composition), can enhance nitrogen assimilation via specialized nitrogen-fixing capabilities, leading to more protein accumulation in the seed than lipids. Our findings align with previous studies in the literature [8,13]. In contrast, Illingworth et al. [37] and Arise et al. [15] reported higher protein compositions of 39.04 and 28.00 g/100 g in defatted moringa seed and fermented jack bean, respectively. This difference may be due to the removal of fat and the pretreatment of jack bean by fermentation prior to analysis. Studies have shown that removal of fat before protein determination, as well as fermentation, can increase the composition and enhance functional properties of protein in food materials [38]. The significant protein content in most of the studied plant sources highlights their potential for food processing in regions where soybean cultivation is limited by unfavorable environmental conditions.

Ash represents the total mineral content of food samples. The ash composition of all the evaluated plant protein sources is comparable to that of soybean, except for rice bran, which exhibited a significantly higher ash content (*p* < 0.05) than soybean. This could be attributed to the high mineral accumulation, as rice bran is the outer layer of the rice grain, where minerals such as phosphorus, magnesium, potassium, and trace elements are more concentrated [5,19]. A study by Fan et al. [39] reported that the removal of the bran layer of rice grain significantly reduced Fe and Zn by 35.2% and 30.2%, respectively, confirming that rice bran is richer in trace mineral elements than the inner endosperm, which primarily stores starch. Additionally, rice plants could have more efficient mechanisms to absorb and store minerals differently than soybeans, partly due to their fibrous root system, which provides a larger surface area that can facilitate efficient absorption of minerals from the soil. The results align with previous reports in the literature [14,40]. Protein-rich plant sources with a moderate ash content can be used for various food applications, while those with higher ash content, such as rice bran, are more suitable for products like bread, crackers, and cookies, where a richer flavor and denser texture are desired [5]. Beyond the reported health benefits of fiber, such as preventing constipation and reducing cholesterol and risk associated with cardiovascular disease [8,41], fiber composition in food ingredients plays a vital role in food processing by influencing texture and satiety. Again, rice bran exhibited the highest fiber content among the plant materials studied, surpassing that of soybean. This can be attributed to the structural composition of rice bran. Studies have shown that rice bran, a protective outside layer of the rice grain, is naturally richer in cellulose, hemicellulose, and lignin than the inner endosperm [41]. The lowest fiber content in sesame is likely due to the high oil accumulation and softer seed structure of sesame, which promote lipid storage rather than structural carbohydrates, as seen in rice bran. Other plant materials had comparable (*p* > 0.05) fiber content to soybean, likely due to similar cell wall compositions and structural characteristics. This suggests that these plant proteins may provide similar texture enhancement in food processing. Our findings align with Maphosa et al. [13] for bambara beans but differ from Beshaw et al. [42], who reported a slightly higher fiber content (4.21 g/100 g) in sesame seeds. This may be attributed to differences in location, soil conditions, and genetic variations, which can influence fiber compositions.

Plant-based fats are generally considered healthy due to their high ratio of unsaturated to saturated fatty acids. The variation in fat composition among the plant sources, compared with soybean, could be attributed to differences in their metabolic mechanisms of fat biosynthesis and storage capacity. For instance, in most oilseeds such as soybean, oil accumulation begins in the plastids [43], where acetyl-CoA is converted into fatty acids via the fatty acid synthase complex. Fatty acids are further converted into triacylglycerols (TAGs), the primary storage form of oil, by the diacylglycerol acyltransferase enzyme (DGAT). Studies have shown that seeds with a high expression of DGAT tend to accumulate more TAGs, leading to increased oil storage [44]. This may explain the higher fat composition observed in moringa seed, sesame seed, and rice bran compared to soybean. This increased fat composition may not necessarily pose a risk of cardiovascular diseases, as plant-derived fats predominantly consist of unsaturated fatty acids, which are known for their health benefits [45]. Our findings are consistent with previous studies in the literature [27,40]. However, Anyiam et al. [18] reported a lower fat composition of 24.81% in fermented moringa seed grown in Nigeria, likely due to the activities of fermenting microorganisms, which hydrolyze fat into fatty acids and glycerol as an energy source. These findings suggest that, in addition to the appreciable protein content, rice bran, moringa seed, and sesame seed could also serve as alternative fat sources to soybean in food processing, offering comparable lipid levels and improving the sensory properties of food. However, to use these plant sources effectively as protein ingredients in food processing, defatting may be required in order to improve the functional properties of the protein. Carbohydrates serve as the primary source of energy in cellular metabolism. The observed variation (*p* < 0.05) in carbohydrate composition among the studied plant protein sources can be partly attributed to differences in other macronutrient compositions. These differences may also be linked to genetic factors, metabolic pathways, and storage priorities, where some seeds allocate more carbon to protein and lipid biosynthesis, while others prioritize starch and non-starch polysaccharide accumulation for energy storage. Our reports align with previous findings in the literature. For example, Purwandari et al. [14] reported carbohydrate compositions of 53.8–63.1 g/100 g in jack bean grown in Indonesia. The composition of a moderate amount of carbohydrates and proteins makes these plant materials suitable for plant-based protein ingredients in energy-rich foods, helping to meet the nutritional needs of active individuals or athletes.

### 4.2. Anti-Nutritional Factors

Plant proteins contain anti-nutritional factors that reduce nutrient bioavailability and protein digestion. The variations in anti-nutritional factors observed in the plant sources, compared with soybean (Table 2), could be attributed to differences in seed structure and environmental conditions affecting the accumulation of anti-nutrients. For instance, phytate levels are influenced by seed mineral storage mechanisms, as phytates act as primary phosphorus reservoirs in plants [46]. The significantly higher phytate content seen in rice bran (*p* < 0.05) compared to soybean could possibly be due to differences in phosphorus uptake mechanisms [19,39]. Our findings align with the report by Leon-Lopez et al. [47], who recorded phytate levels ranging from 13.8 to 16.74 mg/g in moringa seed. The higher oxalate levels recorded in jack bean and sesame seed, compared to soybean, may be attributed to their role in increased calcium regulation for environmental adaptation mechanisms [14]. These seeds may likely accumulate oxalates to chelate excess calcium, forming calcium oxalate crystals for mineral storage as possible adaptation mechanisms to harsh weather. The accumulated oxalates may serve as osmo-protectants, helping plants adapt to nutrient-poor or arid environments. The similarity in tannin content between soybean and most evaluated plant materials suggests comparable levels of phenolic metabolism and secondary metabolite regulation across these species, which contributes to the reported antioxidant properties and health benefits [13]. Excessive consumption of anti-nutrients can impair the bioavailability of proteins and minerals and interfere with digestive enzymes. The permissible levels of oxalate in food are established at 250 mg/100 g, while the tolerable limit for phytate in the human body ranges from 250 to 500 mg/100 g [48]. All six plant protein samples exhibited phytate concentrations exceeding these permissible ranges. The drying temperature, chosen to minimize the loss of essential nutrients, may have been inadequate to significantly reduce these anti-nutritional factors below the permissible limits. To enhance the potential of these plant protein sources in food processing, their anti-nutrient content should be reduced to acceptable levels. This can be achieved through pre-treatment methods such as thermal processing, microbial fermentation, or novel techniques like microwave radiation.

### 4.3. Bulk Density, Swelling Capacity, and Water Activity

Table 2 presents the bulk density (BD), swelling capacity (SC), and water activity (Aw) of the plant protein sources compared with soybean. The BD of food material is an important parameter for determining packaging and material handling requirements in food processing. The variation in BD among the six plant protein sources in this study, compared to soybean, may be attributed to differences in their chemical compositions. For instance, studies have shown that proteins with higher fat content or larger particle sizes generally have lower BD due to their ability to trap air, resulting in a less compact material [34]. Additionally, the presence of fibers or other structural components in the plant proteins can influence how tightly the particles are packed together, further contributing to the observed differences in BD. Our result aligns with the BD of three varieties of bambara bean reported by Ramatsetse et al. [27] in South Africa. SC indicates the ability of food material to absorb water and expand, serving as a valuable predictor of starch composition and pasting properties that influence texture and food quality during processing. The variations in SC observed across the protein materials could be attributed to differences in their chemical composition, particularly the polysaccharides and lipid content. For instance, amylopectin plays a key role in the swelling and pasting of starch granules in plant materials, while amylose and lipids tend to slow the process [49]. A greater proportion of long chains in amylopectin has been linked to increased starch swelling. Awuchi et al. [50] found that higher starch content in flour enhances SC, especially when rich in branched amylopectin. The ratio of amylose to amylopectin in starch varies depending on the plant source, which may account for the differences in SC observed among various plant sources in our study. Also, higher lipid content may reduce the SC by creating a hydrophobic barrier to water absorption. This could possibly explain the significantly (*p* < 0.05) lower SC obtained in moringa seed and sesame seed compared to soybean. Aw in food refers to the amount of water available for microbial growth during storage and is a key factor in controlling the rate of spoilage. Microbial growth, including yeast, molds, and pathogens, contributes to food deterioration within the Aw range of 0.6 to 1.0. Hence, the value for Aw in dried food material should be below 0.6 to retard microbial alterations. The Aw obtained in all six plant proteins evaluated falls within this recommended range, thereby making them less prone to microbial spoilage during storage.

### 4.4. Color Attributes

The appearance of food is a key factor in consumer decision-making, as it influences perceptions of freshness and quality. The variations in color properties observed among the plant protein sources compared with soybean could be attributed to differences in color pigment composition, storage compounds, and phytochemical constituents within each seed. For example, the presence of chlorophyll in the mung bean coat may have contributed to the observed greenish hue of the processed plant material, which supports the findings of Wintersohle et al. [51]. Moreover, carotenoids found in moringa seeds, which contribute to the strong antioxidant activity of the seed [52], may impart the yellow tones observed in these seeds. The red variety of bambara bean used in our study has been reported to contain nearly twice the amount of iron as other varieties [13], which may also contribute to the observed yellow-red coloration. Manzoor et al. [5] reported that the total phenolic compounds in rice range from 269.85 to 1214.7 mgGAE/100 g, more than the 115–177 mg/100 g reported for soybean. This also could partly explain the higher yellowish-brown hue observed in rice bran compared to soybean. The observed color change (ΔE) in the processed plant samples, compared to soybean, may be attributed to the extent of heat-induced reactions during seed processing, particularly the Maillard reaction, which involves interactions between proteins and carbohydrates. Also, the oxidation of polyphenol to quinones may have also occurred, which may further contribute to color change. The considerable similarity in brightness and yellowness among the plant protein sources evaluated, compared with soybean, indicates their potential for improving food appearance.

### 4.5. Protein Solubility

Protein solubility is crucial in food processing because it affects the texture, digestibility, and functional properties of protein in a food matrix [53]. The variation in protein solubility across plant protein materials can be attributed to differences in protein structural conformation and amino acid composition. For example, the solubility of bambara bean protein was attributed to its predominant vicilin protein fraction, which is rich in proline, a key amino acid that enhances protein structural flexibility [54]. The low protein solubility observed in jack bean, compared to soybean, could be attributed to its storage protein composition, particularly the high globulin content (72.1%) and low albumin (22.3%) [55]. Globulins are primarily soluble in dilute salt solutions but exhibit low solubility in water, which may explain the reduced solubility observed in jack beans. A similar solubility pattern was described for chickpea, lentil, faba bean, and pea across a pH range of 2–12, with minimum solubility at a pH range of 4–6 [53], presumably the isoelectric point (pI). Previous studies have also reported similar pI for these plant protein sources, ranging from pH 4 to 6 [3,16,19,54,56], which aligns with our results. The reduced solubility of the proteins observed at pH 4 could be due to changes in the biochemical characteristics of the proteins with pH. At the isoelectric point, a protein exists in an electrically neutral state due to the balance of positive and negative charges on its molecular surface. At this stage, hydrophobic interactions between protein molecules predominate, surpassing the hydrophilic and hydration forces generated by charged residues, resulting in minimal solubility and protein aggregation at pH 4. The increased solubility observed at alkaline pH 10 is attributed to protein unfolding, exposing hydrophilic groups and promoting water interactions [53]. Additionally, alkaline conditions may also degrade non-protein components that obstruct solubility.

### 4.6. Protein Patterns

Differences in the molecular weight of protein have been reported to have a direct influence on the biological and functional properties of proteins [54]. The variations observed in the electrophoretic pattern of the plant protein sources could be attributed to differences in protein structure and amino acid composition [57]. For instance, glycinin (11S globulin) and β-conglycinin (7S globulin) are reported as the two major storage proteins in soybeans [7], which play a crucial role in determining the biological properties of soy protein. Dumitrascus et al. [58] demonstrated that glycinin consists of polypeptides with lower molecular weights ranging from approximately 19 to 35 kDa, while β-conglycinin comprises higher molecular weight bands ranging between 53 and 75 kDa, which aligns with our findings. Bambara bean was found to contain vicilin (7S globulin) as the major storage protein fraction, with 11S globulin (legumin) as the minor protein [54], with molecular weight ranging between 50 and 80 kDa, similar to 48–75 kDa recorded in our study. The similarity in molecular sizes of major proteins observed in mung bean and bambara bean, when compared to soybean, suggests potential similarities in functional properties. The predominant storage proteins in *Moringa oleifera* seeds consist primarily of high-molecular-weight globulins (53%) and low-molecular-weight albumins with bands often less than 50 kDa [59]. Hence, the two major bands observed in moringa seed in our study with molecular weight at 48–63 kDa may correspond to globulin storage proteins in moringa seed. However, sesame seed proteins displayed few and lower molecular weight bands at 17 and ~48 kDa, which suggests that sesame seed may further differ from soybean protein in terms of biological properties and digestibility. This aligns with the findings of Idowu et al. [57] and Di et al. [29], who reported molecular weights of major protein bands in sesame ranging from 6.5 to 50 kDa. The lower molecular weight of sesame proteins suggests they may be easier to digest and absorb compared to soybean proteins. The lighter intensity of protein bands observed for rice bran in the electropherogram could be attributed to the lower protein content in the bran compared to soybean. Additionally, rice bran had significant amounts of fiber and phytic acid, which may affect protein extraction efficiency during electrophoresis, leading to weaker band intensity. Under reducing conditions with mercaptoethanol, no clear changes in protein patterns were observed across the plant protein sources evaluated except for sesame protein and moringa seed. This may suggest that the proteins in these plant materials may primarily consist of non-covalently linked subunits with minimal disulfide bonding for their structure. The unexpected appearance of higher molecular weight bands (~63–75 kDa) in sesame protein after reduction could be due to the aggregation of subunits upon disruption of intramolecular and intermolecular disulfide bonds. This observation is not in isolation, as it aligns with previous findings in the literature. For example, Wintersohle et al. [51] reported the emergence of higher molecular weight protein bands under reducing conditions (although less dominant) compared to non-reducing. The authors attributed this phenomenon to the structural characteristics of albumins and conformational alterations caused by the disruption of disulfide linkages.

### 4.7. In Vitro Simulated Protein Digestion Pattern

Protein digestibility reflects how effectively gastrointestinal proteases break down ingested protein into amino acids. The observed variation in in vitro protein digestibility (IVPD) values among the evaluated plant-based proteins, compared to soybean, may be influenced by several factors, including the amino acid composition and anti-nutritional factors [26,30]. Surprisingly, sesame seed exhibited the lowest IVPD among the evaluated plant protein sources, despite showing protein bands with a molecular weight of less than 50 kDa under non-reducing conditions (Figure 2) and increased protein solubility, comparable with soybean. This suggests that protein digestibility may also be influenced by other factors, such as the protein structural conformation and mechanisms of interactions with digestive enzymes. These findings align with the report by Sibt-e-Abbas et al. [60] but are lower than the value reported by Calvo-Lerma et al. [61] for fermented sesame seed. This difference may be due to the effects of fermentation, which has been shown to enhance IVPD through the action of fermenting microorganisms. Electrophoretic imaging, which serves as an additional indicator of protein degradation, showed similar digestion patterns for the studied plant protein sources compared with soybean under non-reducing conditions (Figure 3). Pepsin breaks down macro-proteins into polypeptides at the gastric stage, and pancreatin further digests them into smaller peptides at the intestinal stage. The reduced intensity and molecular weight of protein bands after 240 min of digestion with pancreatin enzyme indicate that the tested seed proteins are digestible. This could be attributed to the easy access of proteolytic enzymes in the breakdown of the cell wall of the plant protein sources, which further facilitates better protein release for digestion. Rawdkuen et al. [30] suggest that proteins with a higher molecular weight above 25 kDa may contain more hydrophobic and aromatic amino acids in their sequences, which are highly susceptible to cleavage by pepsin. Thus, the unique digestion pattern observed for sesame and jack bean under the gastric phase is likely due to the presence of aromatic amino acids, which facilitate efficient hydrolysis at the gastric stage compared to soybean.

### 4.8. Amino Acid Profile

Amino acid composition is key to evaluating protein quality. The total essential amino acids (TEAAs) in bambara bean are comparable to the TEAAs found in pea (39.10 g/100 g) and soy proteins (39.77 g/100 g) [7]. The essential-to-total amino acid (E/T) ratio for bambara bean (42.47%) meets the daily requirements for adults (15.0%), children (26.0%), and infants (39.0%) [55]. In contrast, moringa seed (31.17%) does not meet the minimum requirement for infants but fulfills the requirements for children and adults. Additionally, the aromatic amino acids (AAAs) in moringa seed and bambara bean are similar to the AAAs reported for beef (7 g/100 g) and egg protein (9 g/100 g) [34]. AAAs (phenylalanine, tyrosine, and tryptophan) are crucial for protein synthesis, neurotransmitter production, and hormone regulation. The main factor contributing to protein deficiency in low- and middle-income countries is the lack of essential amino acids, particularly lysine, which is deficient in staple cereals like maize, rice, and cassava [62]. This means the bambara bean can serve as a valuable complementary protein source to improve dietary quality and address lysine deficiencies in regions heavily reliant on cereal-based diets. The branched-chain amino acids (BCAAs)—leucine, isoleucine, and valine—in moringa seed and bambara bean are not very far from the 18 g/100 g and 20 g/100 g found in beef and egg, respectively [34]. BCAAs have been reported to have beneficial effects on glucose management by promoting insulin secretion and reducing the postprandial glucose response. The glutamic acid is the primary non-essential amino acid (NEAA) in both moringa seed and bambara bean, comparable to the levels found in soy protein (16.8 g/100 g) and pea protein (21.0 g/100 g) [7]. Although glutamate is considered non-essential, it plays a vital role in brain function and metabolism by regulating ammonia levels and serving as the primary excitatory neurotransmitter. It also acts as a precursor for glutathione synthesis, a key antioxidant that neutralizes reactive oxygen species in the body. Glutamic acid is widely used as a flavoring agent in food due to its umami taste. The appreciable amount of glutamic acid in both moringa seed and bambara nut makes them valuable ingredients for flavoring substances in food products.

### 4.9. Biological Quality of Protein

The EAAI values reflect how well a protein meets human essential amino acid requirements by comparing the essential amino acid content to a reference standard and further providing a measure of biological quality of protein superior to the amino acid score [32], while the PDCAAS method for protein quality determination is a way to assess the ability of proteins to fulfill human requirements of essential amino acids and is therefore recommended for evaluating the protein quality of foods intended for human consumption. The estimated essential amino acid index (EAAI) for moringa seed is 86.26%, while bambara bean scored a higher value of 116.96%. According to Machado et al. [63], a protein is considered of high quality when the EAAI value exceeds 90%, of moderate quality when it falls between 70 and 89%, and of low quality when the EAAI is below 70%. If a sample lacks one or more essential amino acids, the EAAI will be zero, as it cannot support human protein synthesis effectively. The EAAI results indicate that moringa seed falls into the moderate-quality category, while bambara bean exceeds the high-quality protein threshold of 90%. PDCAAS values of 100% (or 1.0) indicate that protein provides an adequate quantity of essential amino acids for children and adults. The EAAI and PDCAAS results show that bambara bean offers a more complete and balanced amino acid profile, making it a superior protein source to moringa seed in terms of protein quality. However, moringa seed, which is still a good protein source due to its high composition, may require supplementation with other proteins to meet optimal amino acid needs.

## 5. Conclusions

This study assessed the nutritional components and digestibility profiles of six potential plant protein sources, comparing them to soybean as a reference. Moringa seed exhibited the highest protein composition, while rice bran and jack bean had significantly lower levels, comparable to soybean. Fat composition varied widely, with moringa and sesame seeds having higher fat content than soybeans. Rice bran recorded the highest fiber and ash content, while sesame seed and moringa seed had a comparable yellowness and brightness to soybean in color analysis. Protein solubility followed the order sesame > soybean > rice bran > bambara bean > moringa seed > mung bean > jack bean at pH 10. Anti-nutritional factors varied, with rice bran and jack bean having the highest phytate and oxalate levels, respectively. In vitro protein digestibility ranked as soybean > bambara, mung bean > moringa seed > rice bran > jack bean > sesame seed. From the comparative analysis carried out, moringa seed and bambara bean emerged as the two most promising alternatives due to their high protein composition, favorable physicochemical properties, and protein digestibility, closely resembling soybean. However, despite the high protein composition and favorable digestibility profile in moringa seed, its EAAI and PDCAAS were low. Bambara bean, with higher IVPD, better EAAI, and PDCAAS values, appears to be a more promising alternative to soybean from a nutritional perspective. This study has some limitations that should be considered when interpreting the findings. The protein quality in this study was assessed using in vitro methods. While in vitro digestibility provides useful estimates, they may not fully capture the complexities of human digestion and absorption in a biological system. Future studies should also focus on in vivo trials on amino acid bioavailability and the performance and consumer acceptability of these protein sources in food formulations to validate their industrial applicability.

## Figures and Tables

**Table 1:** Proximate composition of potential plant protein sources (g/100 g).

Sample	Moisture	Ash	Protein	Fiber	Fat	Carbohydrate
Rice bran	4.80 ± 0.35 ^bc^	9.92 ± 0.10 ^a^	14.98 ± 2.18 ^d^	8.18 ± 0.11 ^a^	18.09 ± 1.83 ^c^	44.01 ± 1.83 ^c^
Bambara bean	6.00 ± 0.51 ^a^	3.14 ± 0.11 ^d^	20.59 ± 1.41 ^c^	5.13 ± 0.34 ^b^	6.41 ± 0.64 ^d^	58.72 ± 2.21 ^b^
Moringa seed	5.10 ± 0.22 ^b^	3.45 ± 0.06 ^c^	30.29 ± 2.20 ^a^	4.72 ± 0.02 ^b^	33.30 ± 0.79 ^a^	23.12 ± 2.15 ^e^
Mung bean	2.34 ± 0.78 ^d^	4.62 ± 0.15 ^b^	24.90 ± 0.08 ^b^	5.47 ± 1.21 ^b^	5.19 ± 1.05 ^d^	57.45 ± 1.12 ^b^
Sesame seeds	6.02 ± 0.39 ^a^	3.49 ± 0.23 ^c^	23.30 ± 1.54 ^b^	2.90 ± 0.10 ^c^	23.05 ± 0.98 ^b^	41.21 ± 2.56 ^c^
Jack bean	3.16 ± 0.07 ^d^	3.11 ± 0.08 ^d^	18.65 ± 0.99 ^c^	3.72 ± 0.22 ^c^	6.70 ± 1.22 ^d^	65.65 ± 1.19 ^a^
Soy bean	4.10 ± 0.54 ^c^	3.38 ± 0.06 ^c^	31.96 ± 1.32 ^a^	5.42 ± 0.68 ^b^	19.64 ± 1.81 ^c^	35.47 ± 3.20 ^d^

Data are expressed as mean ± standard deviation of triplicate values. Mean ± SD followed by different letters within each column are significantly different (*p* ≤ 0.05).

**Table 2:** Physicochemical properties and anti-nutritional properties of potential plant protein sources.

Sample	Physicochemical Properties			Anti-Nutritional Properties		
	Bulk Density (g/mL)	Swelling Capacity (mL/g)	Water Activity	Phytate (mg/g)	Oxalate (mg/100 g)	Tannin (mg/100 g)
Rice bran	0.50 ± 0.0 ^b^	0.27 ± 0.0 ^d^	0.38 ± 0.0 ^f^	37.19 ± 2.81 ^a^	6.50 ± 0.26 ^e^	7.01 ± 0.40 ^e^
Bambara bean	0.53 ± 0.1 ^b^	0.33 ± 0.0 ^cd^	0.54 ± 0.0 ^b^	14.65 ± 1.21 ^d^	8.30 ± 0.29 ^c^	10.79 ± 2.57 ^a^
Moringa seed	0.49 ± 0.1 ^b^	0.25 ± 0.0 ^d^	0.60 ± 0.0 ^a^	19.29 ± 2.97 ^c^	9.71 ± 1.09 ^b^	6.64 ± 0.17 ^e^
Mung bean	0.69 ± 0.0 ^a^	0.55 ± 0.0 ^b^	0.46 ± 0.0 ^e^	28.60 ± 1.10 ^c^	7.39 ± 0.53 ^d^	9.50 ± 0.77 ^b^
Sesame seed	0.74 ± 0.1 ^a^	0.26 ± 0.1 ^d^	0.48 ± 0.0 ^d^	13.47 ± 2.92 ^d^	10.10 ± 0.52 ^b^	7.76 ± 0.57 ^cd^
Jack bean	0.64 ± 0.0 ^a^	0.44 ± 0.1 ^bc^	0.50 ± 0.0 ^c^	32.47 ± 2.29 ^b^	12.56 ± 0.44 ^a^	7.16 ± 0.20 ^de^
Soy bean	0.70 ± 0.0 ^a^	0.71 ± 0.1 ^a^	0.50 ± 0.0 ^c^	14.96 ± 0.73 ^d^	7.46 ± 0.16 ^d^	8.11 ± 0.60 ^c^

Data are expressed as mean ± standard deviation of triplicate values. Mean ± SD followed by different letters within each column are significantly different (*p* ≤ 0.05).

**Table 3:** Color attributes of processed flour of potential plant protein sources.

Sample	Appearance	L*	a*	b*	WI	YI	ΔE
Rice bran	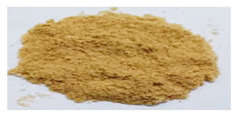	76.37 ± 0.52 ^d^	3.36 ± 0.06 ^a^	20.72 ± 0.01 ^a^	68.38 ± 0.39 ^f^	38.76 ± 0.25 ^a^	19.33 ± 0.52 ^a^
Bambara bean	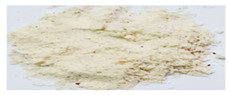	89.09 ± 1.07 ^c^	1.23 ± 0.16 ^b^	8.11 ± 0.21 ^f^	86.33 ± 0.88 ^ab^	13.07 ± 0.42 ^g^	9.61 ± 0.41 ^c^
Moringa seed	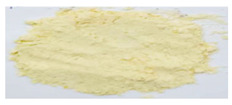	87.97 ± 0.47 ^c^	0.77 ± 0.01 ^c^	13.44 ± 0.53 ^c^	81.94 ± 0.59 ^c^	21.83 ± 0.91 ^d^	18.55 ± 0.61 ^a^
Mung bean	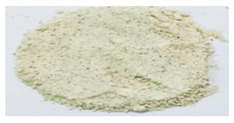	90.90 ± 1.39 ^b^	-0.30 ± 0.07 ^f^	10.95 ± 0.47 ^d^	85.71 ± 0.77 ^b^	17.21 ± 0.66 ^e^	13.01 ± 0.57 ^b^
Sesame seed	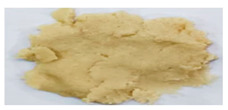	76.96 ± 0.59 ^d^	0.71 ± 0.03 ^c^	18.81 ± 0.64 ^b^	70.23 ± 0.14 ^e^	34.91 ± 0.93 ^b^	10.44 ± 0.13 ^c^
Jack bean	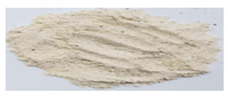	91.63 ± 0.12 ^b^	0.49 ± 0.05 ^d^	9.70 ± 0.09 ^e^	87.17 ± 0.03 ^a^	15.12 ± 0.13 ^f^	9.73 ± 0.90 ^c^
Soy bean	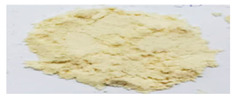	95.14 ± 1.31 ^a^	0.16 ± 0.11 ^e^	19.43 ± 0.14 ^b^	79.88 ± 0.44 ^d^	29.24 ± 0.62 ^c^	NA

Data are expressed as mean ± standard deviation of triplicate values. Mean ± SD followed by different letters within each column are significantly different (*p* ≤ 0.05). NA: not applicable. WI: whiteness and YI: yellowness. ΔE: change in color.

**Table 4:** Amino acid composition of moringa seed and bambara bean (g/100 g crude protein).

Amino acid	Moringa Seed	Bambara Bean	FAO/WHO Reference
Essential amino acids			Children
Threonine	2.52	4.10	3.40
Valine	3.78	5.36	3.50
Phenylalanine	4.41	5.68	6.30
Isoleucine	3.17	4.12	2.80
Leucine	5.78	7.50	6.60
Histidine	2.43	3.03	1.90
Lysine	2.82	6.57	5.80
Methionine	1.98	1.68	2.50
Tryptophan	0.81	1.03	0.85
Non-essential amino acids			
Serine	3.17	5.89	NA
Aspartic acid	4.76	11.52	NA
Glutamic acid	20.66	14.65	NA
Glycine	4.73	3.92	NA
Alanine	4.02	4.11	NA
Tyrosine	1.65	2.80	NA
Cystine	2.83	0.72	NA
Proline	5.49	3.70	NA
Arginine	13.94	5.60	NA
TAA (g/100 g)	88.99	91.98	NA
TEAA(g/100 g)	27.74	39.07	NA
TNEAA (g/100 g)	66.25	52.91	NA
% TEAA	31.17	42.47	NA
BCAAs (g/100 g)	12.73	12.90	NA
AAAs (g/100 g)	6.87	9.51	NA
AAS (%)	49.31 (Lys)	67.20 (Meth)	NA
PDCAAS%	41.42	58.46	NA
E/T (%)	31.17	42.47	NA
EAAI (%)	86.26	116.96%	NA

TEAA = total essential amino acids, TNEAA = total non-essential amino acids, AAS = amino acid score, PDCAAS = protein digestibility corrected amino acid score. EAAI = essential amino acid index. TAA = total amino acids. E/T = essential-to-total amino acid, BCAAs = branched-chain amino acids, AAAs = aromatic amino acids. NA: not applicable.

## Data Availability

The original contributions presented in the study are included in the article, further inquiries can be directed to the corresponding author.
